# Extreme Heat and Depression Risk Among Older Mexican Americans

**DOI:** 10.1093/geroni/igaf122.545

**Published:** 2025-12-31

**Authors:** Alexandra Holland, Sadaf Milani

**Affiliations:** UTMB Health, The University of Texas Medical Branch, Galveston, Texas, United States; University of Texas Medical Branch, Galveston, Texas, United States

## Abstract

Both Texas and California are experiencing rising average temperatures and more frequent days of dangerously elevated temperatures. Extreme temperatures are linked to many poor health outcomes among those 65+, but less is known about the potential effects of temperature change on the risk of depression among older adults, especially for older Hispanic adults. We aimed to examine the association between extremely high temperatures and depressive symptoms among older Mexican Americans living in Texas or California, using data from Wave 5 (2005) of the Hispanic Established Population for the Epidemiological Study of the Elderly (HEPESE), linked to heat index data (gridMET and National Weather Service) and geocoded to census tract. We used a count of days with a maximum heat index of 1) ≥90 °F (extreme heat) and 2) ≥103 °F (dangerous heat). Elevated depressive symptoms were measured with the Center for Epidemiologic Studies Depression Scale. We used linear regression models to test the association between heat index and depressive symptoms, controlling for sociodemographic characteristics. Our sample (n = 1,701) was 81.6 years old on average (SD: 5.0) and 62.0% female. On average, participants had 9.5 (SD: 9.0) depressive symptoms. In fully adjusted analyses, extreme heat (β: -0.006; 95% CI: -0.016, 0.004) and dangerous heat (β: -0.007; 95% CI: -0.029, 0.014) were not associated with depressive symptoms. Future work will incorporate additional waves of data from the HEPESE to detect the potential effects of changes in temperatures over time, along with the potential effects of other variables (e.g.: SES, neighborhood characteristics).

